# Salt Contents in Fermented Dairy Products: A Strategic Blueprint for Healthier Intake

**DOI:** 10.1002/fsn3.4762

**Published:** 2025-01-15

**Authors:** Ali Massomian, Azadeh Rashidimehr, Fatemeh Mohammadi‐Nasrabadi, Khadijeh Khoshtinat, Fatemeh Esfarjani

**Affiliations:** ^1^ Department of Microbiology and Food Hygiene, Faculty of Veterinary Medicine Lorestan University Khorramabad Iran; ^2^ Food and Nutrition Policy and Planning Research Department, Faculty of Nutrition Sciences and Food Technology, National Nutrition and Food Technology Research Institute Shahid Beheshti University of Medical Sciences Tehran Iran; ^3^ Department of Food Science and Technology, Faculty of Nutrition Sciences and Food Technology, National Nutrition and Food Technology Research Institute Shahid Beheshti University of Medical Sciences Tehran Iran

**Keywords:** fermented dairy products, health policy strategies, salt reduction

## Abstract

This study aimed to estimate the quantity and trends of salt intake from industrial fermented dairy products, develop strategies to reduce salt content, and inform policymakers on promoting public health through healthier dairy options. A cross‐sectional study was conducted on fermented dairy products. Seventy‐nine random samples were selected, and the salt (NaCl %) content was determined by potentiometric titration after sample preparation and homogenization; also samples were analyzed for their moisture (oven drying method). Data analysis involved descriptive statistics and one‐sample *t*‐test. A comprehensive literature review on salt reduction strategies was also performed proposing a model for an optimized low‐salt fermented dairy product. This study found high salt content in many fermented dairy products. Brined cheese had the highest salt level (7.57 g/100 g), while pizza processed cheese had the lowest (1.03 g/100 g). Probiotic yogurts contained less salt (0.29 g/100 g) than regular ones. Other products like doogh (1.04 g/100 g), kefir (0.63 g/100 g), and kashk (2.78 g/100 g) also contributed significantly to salt intake. Most products exceeded recommended salt limits. Consuming just one serving of these products often accounted for a substantial portion of the daily recommended salt intake (WHO: 5 g/day). This research emphasizes the need for reducing salt in fermented dairy products to improve public health. This study highlights the excessive salt content in many fermented dairy products, surpassing recommended daily intake levels. Therefore, to address this public health concern, a multi‐faceted approach is necessary. For this purpose, Policymakers should implement stringent monitoring, enforce food labeling, and develop legislation to reduce salt content. Furthermore, the food industry must innovate to reduce salt while maintaining product quality and taste. On the other hand, consumer education and awareness campaigns are crucial for informed choices. Additionally, further research is needed to understand consumer perceptions and the long‐term impact of salt‐sustainable reduction strategies on dietary habits and public health.

## Graphical abstract

Reducing salt in fermented dairy products to improve public health. Challenging the excessive salt content in many fermented dairy products. Implementation of stringent monitoring, enforcement of food labeling, and development of legislation to reduce salt content by policymakers. Innovation for reducing salt methods, also maintaining product quality and taste by food industry. Consumer education and awareness campaigns for informed choices.

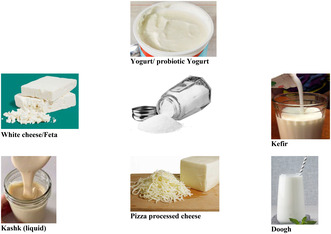

## 1 Introduction

Fermented dairy products, referred to as cultured dairy foods or cultured milk products, encompass dairy items that have undergone fermentation, primarily by *Streptococcus thermophilus* and lactic acid bacteria (LAB) such as *Lactobacillus delbrueckii subsp bulgaricus* (FAO 2011; Savaiano and Hutkins 2021). This fermentation process not only extends the shelf life of these products but also enhances their flavor and digestibility, as well as relieving hypertension (Song et al. 2024). In Iran, a diverse array of fermented dairy products is available, including cheese, yogurt, doogh, kefir, and kashk. In 2021, the *per capita* weighted mean of dairy product consumption averaged 68.34 kg including milk, yogurt, and cheese at 22.68, 11.06, and 3.79 kg, respectively (Roustaee et al. 2024). The processing of traditional fermented foods, such as cheese, yogurt, doogh, kefir, and kashk, has a long‐standing history. These foods are renowned worldwide for their unique flavor and abundant nutritional value. Fermented foods are considered an essential dietary component (Rezac et al. 2018). Recent discussions have highlighted the varying effects of dairy products based on their fat and sodium content (Lordan et al. 2018). Fermented dairy products are known for their complexity, offering high‐quality proteins, minerals, and complex fats. They are a good source of probiotics, the healthy bacteria that may aid the gut, and improve the immune system. The most abundant microbes in them are *Streptococcus* and *Lactobacillus* (Shahrajabian and Sun 2023). A causal link has been established between the digestion and tolerance of lactose and the consumption of yogurt. Additionally, there are consistent correlations between the intake of fermented milk and a decreased risk of breast and colorectal cancers, type 2 diabetes, enhanced weight management, as well as improved cardiovascular, bone, and gastrointestinal health. Moreover, a relationship has been identified between the incidence of prostate cancer and the overall consumption of dairy products, with no distinction made between fermented and unfermented varieties (Savaiano and Hutkins 2021).

Salt (sodium chloride, NaCl) is an inorganic compound composed of sodium and chloride ions (Food and Malaysia 2010). It is necessary for normal body function and food preservation, and it acts as a natural preservative, extending the shelf life of food products while preventing the growth of bacteria and other harmful microorganisms (Rosma et al. 2009).

Cardiovascular diseases are the leading cause of death in Iran, despite being ranked third in terms of the overall burden of disease (Naghavi et al. 2009). There are several risk factors contributing to the increased occurrence of hypertension, such as unhealthy eating habits (mainly consuming excessive salt, saturated fat, and other unhealthy foods), sedentary lifestyle, weight increase, and stress (WHO 2013). In industrialized nations, processed foods account for 75%–80% of dietary salt intake, while naturally occurring salt is found in 5%–10% of the foods consumed, and the remaining 10%–15% is added salt from cooking or the table. In developing nations, on the other hand, salt used for seasoning is far more important (Kloss et al. 2015).

However, consuming too much salt on a regular basis causes conditions like high blood pressure, heart disease, and stomach cancer (Moslemi et al. 2020). One element of salt, sodium, can potentially pose health risks, particularly concerning blood pressure. Studies indicate that a high‐sodium diet is directly linked to hypertension, thereby increasing the risk of cardiovascular diseases (Ayyash and Shah 2010). For example, the primary cause of cardio‐metabolic disorders was a daily sodium intake of 3480 mg, or 8.8 g of salt. A study by the World Health Organization (WHO) reveals that heart conditions cause nearly 17 million deaths worldwide annually (Organization, 2011).

In the latest guidance, the WHO has recommended that countries reduce their salt intake by up to 30% by 2025, and, if possible, aim to limit it to 5 g per day (equivalent to about 2 g of sodium) (WHO 2022).

However, the evidence indicates that Iranians consume two to three times more than the WHO recommended level (Nosratinia et al. 2024; Organization, 2012; Rezaei et al. 2018). A meta‐analysis revealed that reducing salt intake to around 2–3 g per day was linked to a 20%–30% decrease in cardiovascular disease incidence (He, Li, and MacGregor 2013; He and MacGregor 2011; Taylor et al. 2011).

The two national surveys on food consumption and nutritional status of Iranian households between 2000 and 2020 showed that although yogurt consumption decreased (73–48) and doogh consumption increased (9–40) (g/daily *per capita*), the total intake of fermented dairy products and especially cheese (15 g/daily *per capita*) has not changed (NNFTRI 2017).

To our knowledge, this is the first study on salt content in industrial fermented dairy products to develop a strategic plan analysis to propose practical solutions for the development of industry‐healthier fermented dairy products, and facilitate policymakers’ decisions to promote public health.

## 2 Materials and Methods

### 2.1 Phase I

#### 2.1.1 Sampling of Fermented Dairy Products

A cross‐sectional study was conducted on popular brands of industrial fermented dairy products from five distinct food chain stores, including 79 randomly selected samples of cheese, yogurt, doogh, kefir, and kashk in Tehran, Iran (2024). The Iranian Dairy Product Association's marketing unit provided the list of the most popular samples. To transfer the samples to the lab for analysis, they were packaged in a cold environment and coded with a two‐digit number. The samples were produced and promptly refrigerated to 4°C.

#### 2.1.2 Moisture Content

Moisture assays can be one of the most important analyses performed on a food product. This method involves the use of an oven‐drying process (102°C ± 5°C) and, for cheese samples, dried sea sand with constant weight was used. Samples (2–3 g) were weighed into a pair of clean, dried, pre‐weighed aluminum dishes in a moisture dish with a tight‐fitting cover containing sea sand and dried until the weight was constant for two consecutive readings (AOAC 2012; Mauer 2024).

#### 2.1.3 Analysis of Fermented Dairy NaCl

At first, a section of the sample is submerged in water. Three grams of the sample were measured using an analytical scale (EK6100i, A&D, Japan) and added to 30 mL of distilled water that had been heated to 55°C. Using a glass stick, the sample pieces were dissolved, and the suspension was completely homogenized using a mechanical homogenizer (T25 digital ultraturrox, IKA, Germany). Then, 3 mL of a nitric acid solution (4 mol/L) was added to the homogenized sample. Potentiometric measurements were performed with a pH/mV‐meter (Zagshimi, Iran) at 25°C. The Ag/Ag electrode (Azar electrode, Iran) was selected as a reference electrode. During titration, the concentration of chloride ions in the solution decreases as it forms silver chloride, causing the electrode potential to increase until it is fully titrated. Potentiometric titration curves were drawn from the calibrating plot to determine the chloride ion content in dairy product samples. The method used in this study was validated for specificity, linearity, accuracy, precision, and verification to ensure that the results are reproducible and repeatable. The titration curve was obtained by correlating the ion‐selective electrode potential with the volume of added AgNO_3_. The volume of AgNO_3_ at the equivalence point can be determined via the complicated graphical method, and the analysis was performed in triplicate for accuracy.

The following formula was used to determine the mass fraction, in percent, of NaCl:

$$\%\text{NaCl}=\frac{\left(v_{1}-v_{0}\right)\times \frac{c}{1000}\times 58.4}{m}\times 100\%$$

The volume of the AgNO_3_ standard solution in the blank test is denoted by *V*_*0*_ (mL), while *V*_*1*_ (mL) represents the volume of the solution used in the determination. *C* signifies the actual concentration in moles per L (≈1000 mL) of the AgNO_3_ standard solution, and *m* stands for the mass in grams of the test portion.

#### 2.1.4 Calculation of NaCl Intake From Fermented Dairy Products (Serving Size)

The FDA sets the serving size, also known as the standard portion size, for each food item based on how much a person would normally eat in one sitting. It does not provide all the information on what one should eat for a healthy diet, nor is it necessarily the amount that is best for anyone. A serving from the dairy food group is a cup (250 g) of milk, two‐thirds of a cup (180 g) of yogurt, and two slices (30 g) of cheese types. However, the Dietary Guidelines for Americans recommends that adults limit sodium intake to less than 2300 mg per day, which is equal to about one teaspoon of table salt. The salt content of fermented dairy products was calculated to estimate the minimum amount of salt consumed per day by this dairy group (Raymond and Morrow 2022).

### 2.2 Phase II

#### 2.2.1 Salt Reduction Strategies

A comprehensive search was conducted using specific subject headings and keywords related to databases to identify salt reduction strategies, challenges, and potential opportunities using electronic databases, including PubMed, Scopus, Web of Science, ScienceDirect, and Google Scholar. Terms such as “salt intake,” “salt reduction,” “salt strategies,” “dietary strategies,” “dietary guidelines,” and “fermented dairy products” alone or combined by “OR” and/or “AND” were used. In addition, a manual search was performed to strengthen the search strategy, covering study sources and field reviews (Etebarian et al. 2024). Additionally, we collected and analyzed relevant data and statistics from reports by the World Health Organization (WHO) and the Iranian National Standard Organization. It highlighted current trends and global challenges, which helped us gain a comprehensive understanding of the issue and its impact on the global community.

#### 2.2.2 Statistical Analysis

Data analysis was conducted using IBM's Statistical Package for Social Sciences (SPSS) software, version 21. The frequency counts and percentages were used to summarize the data for categorical variables, while the mean and standard error were used for quantitative variables. To assess the differences between dairy products in terms of salt content, a one‐sample t‐test was used with a *p*‐value of less than 0.05 was considered significant.

## 3 Results and Discussion

Among different fermented dairy products, the mean salt content is presented in Table 1. Based on the findings, among cheese samples, brined cheese (Lighvan) had the highest amount (7.57 ± 1.08) and pizza processed cheese had the lowest (1.03 ± 0.07) (g/100 g) and also, the lowest moisture was 30.00 ± 1.47 (g/100 g) was in Parmesan cheese.

**TABLE 1:** The mean ± SE salt content of fermented dairy products compared to labels and standards in Iran.

Types of fermented dairy	Sample size	Salt (g/100 g) (mean ± SE)	Nutrition fact labels (%)	Limit (INSO^a^ no.)	Moisture (g/100 g) (mean ± SE)
Cheese					
White cheese/Feta	10	2.40 ± 0.25	2.52	3 (2344)	63.32 ± 0.45
Cream cheese/Labneh	12	1.20 ± 0.07	1.17	< 1.3 (5881)	65.82 ± 0.99
Lactic cheese	4	3.32 ± 0.25	2.74	< 3.5 (13863)	63.95 ± 0.38
Brined cheese (Lighvan)	6	7.57 ± 1.08*	4.33	< 4.5 (2344‐1)	59.02 ± 0.40
Cheddar cheese	4	1.40 ± 0.09	1.15	< 2 (11832)	49.50 ± 2.98
Pizza processed cheese	10	1.03 ± 0.07*	1.11	< 0.8 (13526)	58.97 ± 5.55
Parmesan cheese	5	2.01 ± 0.61	1.80	< 2.5 (9011)	30.00 ± 1.47
Yogurt					
Yogurt	5	0.36 ± 0.07	0.26	< 0.25 (695)	77.79 ± 4.18
Probiotic yogurt	4	0.29 ± 0.09	0.15	0 (11325)	87.63 ± 1.01
Dairy beverages					
Doogh	6	1.04 ± 0.04*	0.78	< 0.6 (2453)	82.71 ± 4.92
Kefir	6	0.63 ± 0.04	0.50	< 0.7 (11177)	94.63 ± 0.41
Kashk (liquid)	7	2.78 ± 0.25*	2.09	< 3 (2452)	88.96 ± 0.48

*Note:* Each value is the mean ± SE from three independent experiments.

^a^ Iran National Standards Organization.

* Based on a one‐sample *t*‐test, there was a statistically significant difference between brined cheese (lighvan), pizza processed cheese, doogh, kashk (liquid), and INSO limits (*p* < 0.05).

In yogurt samples, probiotics (0.29 ± 0.09) were lower in salt than the regular yogurts. Moreover, doogh (a traditional Iranian drink), kefir, and kashk (liquid) had 1.04 ± 0.04, 0.63 ± 0.04, and 2.78 ± 0.25 (g/100 g), respectively. There was a statistically significant difference between brined cheese (Lighvan), pizza processed cheese, doogh, kashk (liquid), and INSO limits (*p* < 0.05).

**FIGURE 1:**
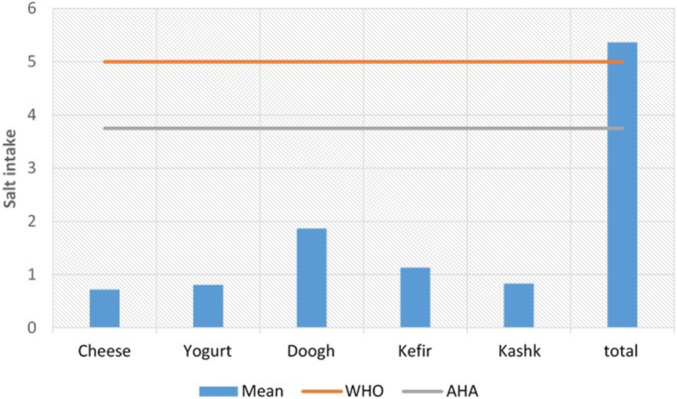
Minimum salt intake from a serving of cheese, yogurt, doogh, kefir, and kashk compared to the recommended daily salt intake by WHO and the American Heart Association (AHA).

As shown in Figure 1, the minimum salt intake from one serving of cheese, yogurt, doogh, kefir, and kashk was importantly noted compared to the recommended daily salt intake of WHO and the American Heart Association (AHA) (Hirose, Tran, and Yamamoto 2021).

While one serving of each product in this specific food group did not exceed the recommended daily allowance, if consumed together during 24 h, the limits could be exceeded.

Therefore, this group is a high priority for policymakers to consider new strategies to reduce salt content. In a study conducted in Isfahan, Iran, it was found that adults consumed an average of 10.9 ± 3.4 g/day, while children and adolescents consumed an average of 10.3 ± 2.9 g/day. The study also highlighted cheese as one of the primary sources of salt in their diets (Mohammadifard et al. 2021). In a review study and systematic meta‐analysis conducted to estimate the average salt intake in the Iranian population without a time limit until the end of 2020, data from 32 studies showed that the average salt consumption in all age groups was 9.674 g/day for all ages. The research results revealed that salt intake is high in the southern to northwestern regions of Iran. Among the provinces, Lorestan individuals consumed the highest amount of salt at 13.05 g/day, while those in Bushehr consumed the least at 8.16 g/day (Pourkhajoei et al. 2022). Individuals in these regions may have different dietary patterns and food diversity due to Iran's large size and diverse population (Rezaei et al. 2018).

Additionally, the studies revealed that the amount of salt consumed in Iran is twice the recommendation of the World Health Organization (Mohammadifard et al. 2012; Pourkhajoei et al. 2022). The variation in average salt intake across studies can be anticipated due to the use of diverse measurement methods. Despite the use of different approaches to determine average salt intake (such as 24‐h urine collection, spot urine samples, overnight urine samples, and Food Frequency Questionnaire) (Pourkhajoei et al. 2022), all the data indicate that salt consumption surpasses the World Health Organization's recommendation for preventing noncommunicable diseases. One reason for consuming too much salt is the lack of knowledge about where it comes from and how much is consumed by individuals, leading to the highest death rate in countries with measures to prevent noncommunicable diseases (Layeghiasl et al. 2020; Loloei et al. 2019).

Several million deaths worldwide could be prevented each year through a reduction in global salt consumption (Chiu, Venkatakrishnan, and Wang 2020; He et al. 2020). Increased dietary sodium intake is strongly correlated with various health issues, such as hypertension, stroke, cardiovascular diseases, and stomach cancer. Concerns about salt intake and its impact on blood pressure were raised during the Food Standards Agency Salt Campaign in England (Wyness, Butriss, and Stanner 2012). Globally, research suggests that reducing salt intake has led to lower blood pressure and a decrease in cardiovascular diseases (Wilson, Komitopoulou, and Incles 2012; Zhu et al. 2019).

The body's system relies on maintaining a balance of electrolytes, such as sodium, potassium, and chlorine. It is worth noting that the human body does not possess the ability to synthesize sodium and therefore relies on dietary sources to meet its sodium requirements. On the other hand, NaCl is crucial for enhancing food safety, flavor, and texture. The consumption of food accounts for approximately 90% of the sodium that the human body requires (He, Campbell, and MacGregor 2012).

To control non‐communicable diseases, such as those related to salt intake, the European Union and the World Health Organization have pledged to decrease salt consumption worldwide by 30% by 2025 (WHO 2022). Therefore, the food industry's primary goal is to reduce the salt content in foods.

Processing not only extends the shelf life of fermented dairy products, but also enhances their flavor and digestibility. In the course of the fermentation reaction in milk, lactose is often metabolized by LAB to lactic acid, which results in Ph lowering and higher titratable acidity (TA). As a result, pathogen organisms cannot proliferate in fermented dairy products. People's health and the consumption of fermented dairy products are significantly correlated. In Iran, a diverse array of fermented dairy products is available, including cheese, yogurt, doogh, kefir, and kashk. The different climate (at the same time) in this country makes the production of a wide range of dairy‐fermented products possible (Noori et al. 2013).

### 3.1 Cheese

One of the important fermented dairy products is cheese, which is prepared with added salt and has a higher salt content than other milk‐based products. The percentage of salt in cheeses can vary greatly. Consuming high amounts of salty cheese, like Blue, Feta, or Domiati, can significantly contribute to sodium intake in the diet (Guinee and Fox 2017).

In Iran, it is common to eat cheese for breakfast, particularly brined cheese (Lighvan), a traditional Iranian cheese that has a high salt content. Lighvan cheese is a semi‐hard, ripened cheese made from raw sheep milk or, recently, pasteurized milk in Iran.

The process involves curdling milk using commercial rennet, cutting the curd, soaking it in a 24% brine for 24 h, salting the surface, and storing it in caves at 8°C–10°C for 90 days until it reaches the quarantine and ripening period (Navidghasemizad et al. 2009).

Cheese is known for its high sodium content, which is added during the cheese‐making process. Natural cheese typically contains 40–800 mg of sodium per 100 g, while processed cheese can have as much as 1500 mg of sodium per 100 g (Zonoubi and Goli 2021).

To increase their shelf life, they are stored in NaCl brine at a lower pH and refrigerated. This method can increase their shelf life by up to 1–2 months (ISIRI 2015; Nottagh et al. 2020; Omrani Khiabanian et al. 2020).

There is strong evidence that salt contributes to the taste and aroma of cheese and also serves a preservative role. The preservative effect of the salt is specifically linked to its mechanism of lowering water activity (*a*_*w*_). It is believed that the water in cheese that is not contributing to the vapor pressure is retained by the cheese matrix and is therefore not available for microbial activity. Moreover, salt raises the osmotic pressure of the aquatic phase of the food and dehydrates the bacteria.

According to a study conducted in Iran in 2021, bread, cheese, and added salt accounted for over 70% of the total sodium consumed by both age groups. Future nationwide research is necessary to evaluate the amount of salt used in food and its primary sources in the various regions of the Islamic Republic of Iran (Mohammadifard et al. 2021). The negative impacts of salt‐reduction methods on the sensory quality of cheeses have been demonstrated, indicating that these approaches are not yet suitable for implementation. However, they have been subsequently modified and refined to mitigate the effects associated with the salt reduction process (Ferroukhi et al. 2024).

### 3.2 Yogurt

Yogurt is fermented milk that has been shown to include mineral salts such as calcium, zinc, phosphorus, and potassium. Calcium is important for the development of bones and teeth, helping children grow and lowering the risk of osteoporosis in adults (Demirkol and Tarakci 2018; Maharani, Soviana, and Pisestyani 2020). But regular yogurt consumption also has other advantages, such as better protein and sugar digestibility than milk; lactic acid, which stimulates peristaltic movements and aids in digestion; improved oral health beneficial microbial colonization of the gastrointestinal tract; support system development and maintenance; immune system stimulation and inflammation reduction; increased synthesis of hormones and enzymes; simpler absorption of mineral salts; and so on (Ribeiro et al. 2018).

Yogurt is understood as the product resulting from the fermentation of pasteurized or sterilized milk, with fermentation carried out using protosymbiotic cultures of *Streptococcus salivarius subsp*. *Thermophilus* and *Lactobacillus delbrueckii subsp. Bulgaricus* (Tewari, David, and Gautam 2019). Furthermore, yogurt is thought to be an effective means of giving customers probiotic bacteria; it usually contains probiotic cultures in addition to starter germs (Arab et al. 2019). In the case of yogurt, starter cultures do not survive gastrointestinal conditions or colonize the human gut, making them unlikely to provide therapeutic benefits (Fazilah et al. 2018). Because probiotics are live microorganisms that can enter the gastrointestinal system and improve a person's health, they are added to yogurt starter cultures in order to offer functional benefits to humans (Fazilah et al. 2018).

### 3.3 Fermented Traditional Beverages

Fermented traditional drinks are an essential component of diets around the world and are good for human health. Numerous fermented foods enjoyed by various ethnic groups have medicinal properties.

Middle East is the origin of different and traditionally fermented dairy products. Several studies on food fermentation have demonstrated the importance of these products due to their ability to inactivate or decompose anti‐nutritive components and toxins, as well as improve digestibility of nutrients (Mathara et al. 2008).

#### 3.3.1 Kefir

One of the popular fermented dairy products that has gained a lot of attention recently is kefir. Kefir is known for its unique flavor and probiotic properties; this product is produced by fermenting milk with kefir grains. Kefir is rich in probiotics, which are beneficial for gut health. The fermentation process results in the production of lactic acid, which helps in preserving the product and contributes to its tangy flavor. The probiotic strains present in kefir, including various *Lactobacillus* and *Pediococcus species*, have been shown to survive gastric acidity and bile salts, enhancing their potential health benefits (Doğan and Ay 2021; Taheur et al. 2023). The salt content in kefir can vary depending on the production method and the specific recipe used. Generally, kefir is considered to have low to moderate salt content. The presence of salt in kefir is not a primary characteristic but can be influenced by the addition of salt during the fermentation process or by the type of milk used.

#### 3.3.2 Doogh (Yogurt Drinking)

According to codex standard, doogh/ayran is a traditional dairy product. It is produced by the fermentation of semi‐skimmed or whole milk with yogurt bacteria, reconstitution of approximately 35%, and salt fortification of approximately 1% (ISIRI 2015; Noori et al. 2013). The research also indicated that the lactation period notably impacts the chemical and physical characteristics of yogurt (Mohammadifard et al. 2012).

### 3.4 Kashk (Liquid)

Kashk is a dairy product originating from the Middle East, available in various forms (circular, oval, and conical shapes). Traditionally made from raw sheep milk, it can also be derived from cheese‐making by‐products, such as fermented, salted, and dried buttermilk. It is found in liquid and dried varieties. Industrial liquid kashk is produced from concentrated yogurt or dry kashk and requires refrigeration. Conversely, dried kashk can be stored at room temperature for extended periods without spoilage, retaining its nutrients. Kashk is a rich source of proteins, B vitamins, minerals including calcium and potassium, and organic acids such as lactic acid (Soltani and Güzeler 2013). The liquid kashk available in the market is made from industrial yogurt from bovine milk and its quality greatly relies on the type of raw materials utilized, be it dried kashk from sheep milk or yogurt from bovine milk (Ezzatpanah 2020). In this study, the amount of kashk salt was 3.4%, which exceeds the recommended standard limit of 2%. Furthermore, other studies showed that liquid kashk contained 3% NaCl (Shiroodi et al. 2012), which aligns with the findings of this study. Compared to the report of the national program in Iran (2016), the total mean has decreased slightly (3.61 ± 2.28–3.42 ± 0.32) (NNFTRI 2017). In a study conducted on traditional kashk from villages around Tehran, the salt content was found to be between 0.89% and 1.52%, which is below the maximum acceptable level(A Noori et al. 2013).

Comparing our data to one of the biggest studies conducted on the content of sugar, salt, fat, saturated, and trans fatty acids in Iran's industrial and guild foods in 2016 (a national report), the salt content of cheese and yogurt slightly increased; however, these amounts were improved in kashk and doogh (NNFTRI 2017).

Comparing our data in (2024) to one of the biggest studies conducted on the content of sugar, salt, fat, saturated, and trans fatty acids in Iran's industrial and guild foods in 2016 (a national report), the salt content of cheese and yogurt was slightly increased; however, these amounts were increased in kashk and doogh (NNFTRI 2017).

### 3.5 Mislabeling in Fermented Products

It is important to take note of Table 1, which provide compelling evidence that the labeled contents of products were consistently lower than the actual amount in most cases. This discrepancy not only misleads consumers but also raises concerns about the accuracy of product labeling, which should be a top priority for all manufacturers.

The lack of comprehensive and robust data is a major challenge for monitoring implementation efforts. Insufficient funding and technical capacity are the primary reasons for this data gap. Identifying the primary sources of salt in diets is essential for targeting impactful interventions and establishing salt reduction targets for foods and meals (Bhat et al. 2020). To achieve this, conducting repeated detailed 24‐h dietary recalls alongside current, context‐specific food composition tables that include sodium content is essential. Since 2009, the Ministry of Health and its related organizations have been working to reduce salt consumption through various strategies, including product labeling (WHO 2010). A large survey of the Iranian population in 2016 revealed that salt consumption had remained constant (Rezaei et al. 2018). One possible reason for this lack of success could be the insufficient monitoring of product labeling.

### 3.6 Part II

#### 3.6.1 Salt Reduction Strategies in Fermented Dairy Products

It is crucial to adopt strategic approaches and reformulation methods to reduce salt in foods, while still maintaining their flavor and quality. According to the literature review of studies, Britain, Finland, and Japan are the most successful countries in the reduction of salt intake (Hashem, Pombo‐Rodrigues, and Capewell 2015; Ji et al. 2012). These nations have succeeded because they have collaborated with the food sector, supported lawmakers, and conducted systematic awareness efforts.

It is essential to note that the successful reformulation of food products hinges on the resulting items being not only healthy, but also of exceptional quality, possessing superior texture, safety, palatability, and cost‐effectiveness (Jiménez‐Colmenero et al. 2010). To achieve this, extensive studies have been conducted on food reformulation, addressing both the technical aspects of reducing sucrose or saturated fat and consumer elements such as food preferences and human behavior (Grunert et al. 2012; Jiménez‐Colmenero et al. 2010).

Reformulating food by removing salt can lead to fatal food poisoning outbreaks. This is because the removal of these ingredients increases the water activity in the food, which creates an environment where bacteria can grow more easily and cause food poisoning (Stones 2014). To prevent this, food manufacturers often need to reduce the shelf life of the food to ensure that bacteria cannot reach harmful levels. Reducing salt levels in food products can impact their safety. Therefore, it is important to understand the eating habits of consumers before reformulating products (Gomes et al. 2023). While reformulation may decrease the intake of certain foods, it can also improve the nutritional value of processed foods by reducing harmful ingredients and increasing essential nutrients like vitamins and minerals. It can also facilitate low‐cost food fortification to improve the nutrient density of diets (Spieldenner and van der Horst 2018a, 2018b).

On the other hand, intrinsic factors such as pH, salt, sugar, and preservatives contribute to food safety and shelf life in a wide range of foods (Komitopoulou and Gibbs 2012). To address the issue of sodium reduction, food industries have had to find effective replacements for salt capacity. Additionally, the industry is working on obtaining healthier nutrients by using alternative ingredients that replace saturated fats while maintaining the structure of the food. However, it is important to consider whether the substitutes used are necessarily better for health than the original ingredients.

Governments and the food industry are working on reducing sodium in processed foods. While sodium plays a role in maintaining osmotic balance and pH levels, excessive consumption can result in fluid retention and elevated blood pressure (Ekaterina and Feng 2013).

Telling customersthat they consume too much salt and expecting them to alter their eating habits is the most straightforward approach to reducing sodium intake. Diets low in sodium are hard to stick to because they call for a shift in eating habits, such as selecting low‐sodium items that are hard to find (Bilyaminu 2013; Dötsch et al. 2009). In a study conducted in Yasuj, Iran, an intervention based on the social marketing model was designed and implemented to decrease salt consumption. The average salt intake among the study population was almost three times higher than the level recommended by the World Health Organization (WHO). Qualitative findings revealed that most participants acknowledged the high salt consumption in Iran. Furthermore, they suggested home and family‐based strategies to reduce salt intake, recommended the use of healthier alternatives to salt, and identified doctors and healthcare providers in health centers as the most important influencers in encouraging people to reduce salt intake. The intervention, based on social marketing, successfully reduced the salt consumption of the study subjects by about 3 g (Layeghiasl et al. 2020).

There are many ways to reduce salt intake; substituting sodium with a variety of substances that produce a similar level of saltiness when ingested would be excellent (Liem, Miremadi, and Keast 2011). Using taste enhancers (e.g., peptide enhancers) to stimulate salt taste receptors is an additional method of reducing salt intake. These ingredients are added to recipes to enhance the flavor (Le et al. 2022).

Many strategies for reducing salt have also been developed recently. These include reducing salt gradually by altering the structure of sodium chloride, reducing salt gradually over time, and using emerging technologies like altering the food matrix or encapsulating salt in emulsions (Wallis, Chapman, and Campden 2012).

There are currently different methods for achieving a salty taste while reducing sodium content: to influence taste receptors or signaling pathways with unsalted substances, although this method is still only theoretical at this point (Bansal and Mishra 2020). This can be achieved by partially or fully substituting NaCl with other salts such as potassium chloride, magnesium chloride, or calcium chloride (Sebranek 2015). This can be achieved by using salt enhancers such as amino acids and hydrolysates of protein, nucleotides, spices, herbs, and seaweed (Rysová and Šmídová 2021).

Despite significant variances in different scales, the results of a study conducted in Iran showed that high salt intake was a prominent risk factor in all regions of the country. Iran's public health issue requires more appropriate population‐wide measures to address it (Azadnajafabad et al. 2021).

To create healthy food policies and meet national and international goals, the public sector, private health groups, and the food industry must work closely together. Kashk, doogh, and cheese were the major sources of salt in daily dietary intake. For these products, no new standards have been created. The prevention of NCDs is aided by a variety of treatments and variables, such as food reformulation, labeling, monitoring, public awareness campaigns, and marketing. Dietary patterns may be adversely affected by socioeconomic variables, such as price inducement and advertising that incentivizes choosing larger quantities (Mayen et al. 2014). To maximize milk and dairy options and decrease salt in food baskets for the community, policymakers should prioritize strategies based on challenges and opportunities. Concerns about salt reduction have led to the modeling of several creative ideas for significant consumer salt intake reductions and the development of appropriate techniques that pose no health hazards, thanks to the efforts of several governments and other stakeholders in the food sector.

#### 3.6.2 Salt Reduction Implementing Strategies

##### 3.6.2.1 Policymakers

- *Monitoring*

More specifically, we will focus on developing an action plan for salt reduction, enforcing food labeling to include salt content, and developing legislation for monitoring salt reduction implementation.

− Governments need to consider potential policy measures, and more programs are needed to raise consumer awareness.
− Creating a national standard for the salt content of fermented dairy products.
− Salt menu labeling of foods sold in schools, hospitals, cafes, and restaurants.
− Leadership and regulatory processes are essential for prioritizing and driving action on salt reduction across different areas. Effective leadership involves allocating funds, seeking technical support, and establishing policies or regulations.
− Promote the food industry to reduce salt content (tax reduction as an incentive).

b *Reformulation*

All the reformulation initiatives were led by governmental authorities while engaging with the food industry.

c *Consumer awareness*

Increasing consumer knowledge and education around salt consumption in food is a part of most methods put into practice. Academicians lead this aspect of the awareness campaign, while the government spearheaded the majority of it. Public ads, media interviews, and instructional initiatives at schools were all part of the campaign.

##### 3.6.2.2 Food Industry

The producers need to be cautious when using salt to replace components of items with less salt. The two major methods for lowering the amount of salt in cheese are to use salt‐replacement agents in place of sodium chloride salt, providing the flavor of saltiness, enhancing or masking other flavors, and controlling the growth of microorganisms.

− Innovative and emerging technologies in salt reduction, such as changing the food matrix and encapsulating salt in emulsions.
− Replace salt with other flavorings (common salt substitutes, including potassium chloride, certain herbs, spices, organic acids, autolyzed yeast products, and hydrolyzed vegetable protein products alone or in combination).
− Influence taste receptors or signaling pathways with unsalted substances.
− Using taste enhancers is an additional method of reducing salt intake.
− Social marketing is necessary to affect consumers' behavior in reducing salt intake.
− Finding natural salt replacements that are nontoxic and metabolizable.

##### 3.6.2.3 Consumers

− Choose low‐sodium products (sodium‐free or salt‐free, very low sodium, low sodium, reduced or less sodium, lite or light in sodium, unsalted or no salt added).
− Check the nutrition label and the ingredients include salt or items that contain sodium: Monosodium glutamate (MSG), baking soda, also called sodium bicarbonate, baking powder, disodium phosphate, sodium alginate, sodium citrate, and sodium nitrite.

## 4 Future Research

More studies and propaganda about fermented dairy products are necessary, which may influence the Western diet and decrease the high consumption of salt. Research must be carried out on consumer perceptions to better understand the effectiveness of this policy. To devise a strategy for reducing salt in the food supply, formative research is essential. Future research should focus on developing more efficient methods for rapid diet assessment, considering regional variations to pinpoint areas for targeted salt reduction programs. Establishing comprehensive databases, supported by laboratory analysis, for compliance with labeling requirements is critical for effective monitoring.

Lack of reliable data on sources of salt intake in the fermented dairy group of the Iranian diet for evidence‐informed salt reduction policymaking is a major limitation.

## 5 Conclusion

This study reveals alarmingly high salt levels in a variety of popular fermented dairy products, particularly in brined cheese (Lighvan). The majority of products exceeded both nutritional guidelines and national standards. Probiotic yogurts demonstrated lower salt content compared to regular varieties. Notably, a single serving of these products often contributed significantly to the daily recommended salt intake (WHO: 5 g/day). These findings underscore the urgent need for comprehensive strategies to reduce salt content in fermented dairy products.

Policymakers must implement stringent monitoring, enforce food labeling, and develop legislation to reduce salt content and also encourage the food industry to reformulate food products. Consumers should consider behavioral modifications to reduce their risk of NCDs resulting from their diet. The food industry must innovate to reduce salt while maintaining product quality and taste. Consumer education and awareness campaigns are crucial for informed choices. Further research is needed to understand consumer behavior, effective communication strategies, and the impact of salt reduction on overall diet and health. Collaborative efforts between governments, industries, and consumers are essential to achieve significant and sustainable reductions in salt intake from fermented dairy products.

## Author Contributions

**Ali Massomian:** conceptualization (equal), data curation (equal), methodology (equal), software (equal), writing – original draft (equal), writing – review and editing (equal). **Azadeh Rashidimehr:** conceptualization (equal), methodology (equal), project administration (equal), software (equal), writing – original draft (equal). **Fatemeh Mohammadi‐Nasrabadi:** conceptualization (equal), data curation (equal), methodology (equal), software (equal), validation (equal), writing – original draft (equal). **Khadijeh Khoshtinat:** conceptualization (equal), data curation (equal), methodology (equal), resources (equal), writing – original draft (equal). **Fatemeh Esfarjani:** conceptualization (equal), data curation (equal), formal analysis (equal), funding acquisition (equal), investigation (equal), methodology (equal), project administration (equal), resources (equal), software (equal), supervision (equal), validation (equal), visualization (equal), writing – original draft (equal), writing – review and editing (equal).

## Ethics Statement

IR.SBMU.nnftri.Rec.1402.008.

## Conflicts of Interest

The authors declare no conflicts of interest.

## Data Availability

The data will be available upon request from the corresponding authors.

## References

[fsn34762-bib-0001] AOAC . 2012. Official Methods of Analysis. Gaithersburg: AOAC International.

[fsn34762-bib-0002] Arab, M. , S. Sohrabvandi , N. Khorshidian , and A. M. Mortazavian . 2019. “Combined Effects of Salt‐Related Variables on Qualitative Characteristics of Probiotic Fermented Milk.” Current Nutrition & Food Science 15, no. 3: 234–242.

[fsn34762-bib-0003] Ayyash, M. M. , and N. P. Shah . 2010. “Effect of Partial Substitution of NaCl With KCl on Halloumi Cheese During Storage: Chemical Composition, Lactic Bacterial Count, and Organic Acids Production.” Journal of Food Science 75, no. 6: C525–C529. 20722906 10.1111/j.1750-3841.2010.01691.x

[fsn34762-bib-0004] Azadnajafabad, S. , N. Ebrahimi , E. Mohammadi , et al. 2021. “Disparities and Spatial Variations of High Salt Intake in Iran: A Subnational Study of Districts Based on the Small Area Estimation Method.” Public Health Nutrition 24, no. 18: 6281–6291. 34261565 10.1017/S1368980021002986 PMC11148577

[fsn34762-bib-0005] Bansal, V. , and S. K. Mishra . 2020. “Reduced‐Sodium Cheeses: Implications of Reducing Sodium Chloride on Cheese Quality and Safety.” Comprehensive Reviews in Food Science and Food Safety 19, no. 2: 733–758. 33325171 10.1111/1541-4337.12524

[fsn34762-bib-0006] Bhat, S. , M. Marklund , M. E. Henry , et al. 2020. “A Systematic Review of the Sources of Dietary Salt Around the World.” Advances in Nutrition 11, no. 3: 677–686. 10.1093/advances/nmz134. 31904809 PMC7231587

[fsn34762-bib-0007] Bilyaminu, I. B. 2013. “Safety and Quality Aspects of Reducing Salt Content in Foods.” International Journal of Current Research and Review 5, no. 20: 60–65.

[fsn34762-bib-0008] Chiu, H. F. , K. Venkatakrishnan , and C. K. Wang . 2020. “Nutraceuticals and Functional Foods in the Prevention of Hypertension Induced by Excessive Intake of Dietary Salt.” In Dietary Sugar, Salt and Fat in Human Health, edited by H. G. Preuss and D. Bagchi , 423–450. Amsterdam, Netherlands: Elsevier.

[fsn34762-bib-0009] Demirkol, M. , and Z. Tarakci . 2018. “Effect of Grape ( *Vitis labrusca* L.) Pomace Dried by Different Methods on Physicochemical, Microbiological and Bioactive Properties of Yoghurt.” Lwt 97: 770–777.

[fsn34762-bib-0010] Doğan, M. , and M. Ay . 2021. “Evaluation of the Probiotic Potential of Pediococcus Strains From Fermented Dairy Product Kefir.” Czech Journal of Food Sciences 39, no. 5: 376–383.

[fsn34762-bib-0011] Dötsch, M. , J. Busch , M. Batenburg , et al. 2009. “Strategies to Reduce Sodium Consumption: A Food Industry Perspective.” Critical Reviews in Food Science and Nutrition 49, no. 10: 841–851. 19960392 10.1080/10408390903044297

[fsn34762-bib-0012] Ekaterina, T. , and Z. Feng . 2013. “Dietary Salt Intake and Stroke.” Acta Pharmacologica Sinica 34: 8–9. 23274412 10.1038/aps.2012.179 PMC4086497

[fsn34762-bib-0013] Etebarian, A. , B. Alhouei , F. Mohammadi‐Nasrabadi , and F. Esfarjani . 2024. “Propolis as a Functional Food and Promising Agent for Oral Health and Microbiota Balance: A Review Study.” Food Science & Nutrition 12, no. 8: 5329–5340. 39139934 10.1002/fsn3.4216 PMC11317756

[fsn34762-bib-0014] Ezzatpanah, H. 2020. “Traditional Food and Practices for Health: Iranian Dairy Foods.” In Nutritional and Health Aspects of Food in South Asian Countries, 275–287. USA: Academic Press, Elsevier.

[fsn34762-bib-0015] Fazilah, N. F. , A. B. Ariff , M. E. Khayat , L. Rios‐Solis , and M. Halim . 2018. “Influence of Probiotics, Prebiotics, Synbiotics and Bioactive Phytochemicals on the Formulation of Functional Yogurt.” Journal of Functional Foods 48: 387–399.

[fsn34762-bib-0016] Ferroukhi, I. , J. Dominguez , C. Bord , D. Guerinon , C. Chassard , and J. Mardon . 2024. “How Can the NaCl Content of Ripened Fourme d'Ambert Cheese Be Reduced Using Innovative Dry Surface Salting Processes?” International Journal of Dairy Technology 77, no. 2: 548–558.

[fsn34762-bib-0017] Gomes, A. , A. I. Bourbon , A. R. Peixoto , et al. 2023. “Chapter 9—Strategies for the Reduction of Sugar in Food Products.” In Food Structure Engineering and Design for Improved Nutrition, Health and Well‐Being, edited by M. Â. P. R. Cerqueira and L. M. P. Castro , 219–241. USA: Academic Press, Elsevier.

[fsn34762-bib-0018] Grunert, K. , R. Shepherd , W. Traill , and B. Wold . 2012. “Food Choice, Energy Balance and Its Determinants: Views of Human Behaviour in Economics and Psychology.” Trends in Food Science and Technology 28: 132–142. 10.1016/j.tifs.2012.06.007.

[fsn34762-bib-0019] Guinee, T. P. , and P. F. Fox . 2017. “Salt in Cheese: Physical, Chemical and Biological Aspects.” In Cheese, 317–375. USA: Academic Press, Elsevier.

[fsn34762-bib-0020] Hashem, K. M. , S. Pombo‐Rodrigues , and S. Capewell . 2015. “Reducing Sodium in the Global Food Supply to Reduce Population Burden of Cardiovascular Disease.” Current Cardiovascular Risk Reports 9: 1–6.

[fsn34762-bib-0021] He, F. J. , N. R. Campbell , and G. A. MacGregor . 2012. “Reducing Salt Intake to Prevent Hypertension and Cardiovascular Disease.” Revista Panamericana de Salud Pública 32, no. 4: 293–300. 10.1590/S1020-49892012001000008. 23299291

[fsn34762-bib-0022] He, F. J. , J. Li , and G. A. MacGregor . 2013. “Effect of Longer Term Modest Salt Reduction on Blood Pressure: Cochrane Systematic Review and Meta‐Analysis of Randomised Trials.” BMJ 346: f1325. 23558162 10.1136/bmj.f1325

[fsn34762-bib-0023] He, F. J. , and G. A. MacGregor . 2011. “Salt Reduction Lowers Cardiovascular Risk: Meta‐Analysis of Outcome Trials.” Lancet 378, no. 9789: 380–382. 21803192 10.1016/S0140-6736(11)61174-4

[fsn34762-bib-0024] He, F. J. , M. Tan , Y. Ma , and G. A. MacGregor . 2020. “Salt Reduction to Prevent Hypertension and Cardiovascular Disease.” Journal of the American College of Cardiology 75, no. 6: 632–647. 10.1016/j.jacc.2019.11.055. 32057379

[fsn34762-bib-0025] Hirose, K. , T. P. Tran , and S. Yamamoto . 2021. “Decreasing Salt in Hospital Meals Reduced Energy Intake in Elderly Japanese Inpatients.” Journal of Nutritional Science and Vitaminology 67, no. 2: 105–110. 33952730 10.3177/jnsv.67.105

[fsn34762-bib-0026] ISIRI . 2015. “Milk and Milk Products Fresh Cheese: Specifications and Test Methods.” National Standard 6629.

[fsn34762-bib-0027] Ji, C. , L. Sykes , C. Paul , et al. 2012. “Systematic Review of Studies Comparing 24‐Hour and Spot Urine Collections for Estimating Population Salt Intake.” Revista Panamericana de Salud Pública 32: 307–315. 23299293 10.1590/s1020-49892012001000010

[fsn34762-bib-0028] Jiménez‐Colmenero, F. , T. Pintado , S. Cofrades , C. Ruiz‐Capillas , and S. Bastida . 2010. “Production Variations of Nutritional Composition of Commercial Meat Products.” Food Research International 43: 2378–2384. 10.1016/j.foodres.2010.09.009.

[fsn34762-bib-0029] Kloss, L. , J. D. Meyer , L. Graeve , and W. Vetter . 2015. “Sodium Intake and Its Reduction by Food Reformulation in the European Union—A Review.” NFS Journal 1: 9–19.

[fsn34762-bib-0030] Komitopoulou, E. , and P. Gibbs . 2012. “The Pitfalls of Product Reformulation and How to Avoid Them.” https://www.food‐safles/3763‐reformulation‐the‐pitfalls‐of‐product‐reformulation.

[fsn34762-bib-0031] Layeghiasl, M. , J. Malekzadeh , M. Shams , and M. Maleki . 2020. “Using Social Marketing to Reduce Salt Intake in Iran.” Frontiers in Public Health 8: 207. 10.3389/fpubh.2020.00207. 32582611 PMC7289950

[fsn34762-bib-0032] Le, B. , B. Yu , M. S. Amin , et al. 2022. “Salt Taste Receptors and Associated Salty/Salt Taste‐Enhancing Peptides: A Comprehensive Review of Structure and Function.” Trends in Food Science & Technology 129: 657–666.

[fsn34762-bib-0033] Liem, D. G. , F. Miremadi , and R. S. Keast . 2011. “Reducing Sodium in Foods: The Effect on Flavor.” Nutrients 3, no. 6: 694–711. 22254117 10.3390/nu3060694 PMC3257639

[fsn34762-bib-0034] Loloei, S. , H. Pouraram , R. Majdzadeh , A. Takian , M. Goshtaei , and A. Djazayery . 2019. “Policy Analysis of Salt Reduction in Bread in Iran.” AIMS Public Health 6, no. 4: 534–545. 10.3934/publichealth.2019.4.534. 31909073 PMC6940570

[fsn34762-bib-0035] Lordan, R. , A. Tsoupras , B. Mitra , and I. Zabetakis . 2018. “Dairy Fats and Cardiovascular Disease: Do We Really Need to Be Concerned?” Food 7, no. 3: 29. 10.3390/foods7030029 PMC5867544 29494487

[fsn34762-bib-0036] Maharani, M. , S. Soviana , and H. Pisestyani . 2020. “Examination of Milk Quality From Milk Shops in the Residential Areas of IPB Dramaga and Cilibende.” Journal Kajian Veteriner 8: 24–33.

[fsn34762-bib-0037] Mathara, J. M. , U. Schillinger , P. M. Kutima , et al. 2008. “Functional Properties of *Lactobacillus plantarum* Strains Isolated From Maasai Traditional Fermented Milk Products in Kenya.” Current Microbiology 56: 315–321. 18175177 10.1007/s00284-007-9084-6

[fsn34762-bib-0038] Mauer, L. J. 2024. “Moisture and Total Solids Analysis.” In Nielsen's Food Analysis, 233–260. Cham: Springer.

[fsn34762-bib-0039] Mayen, A.‐L. , P. Marques‐Vidal , F. Paccaud , P. Bovet , and S. Stringhini . 2014. “Socioeconomic Determinants of Dietary Patterns in Low‐and Middle‐Income Countries: A Systematic Review.” American Journal of Clinical Nutrition 100, no. 6: 1520–1531. 25411287 10.3945/ajcn.114.089029

[fsn34762-bib-0040] Mohammadifard, N. , S. Fahimi , A. Khosravi , et al. 2012. “Advocacy Strategies and Action Plans for Reducing Salt Intake in Iran.” Archives of Iranian Medicine 15, no. 5: 320–324. (Report). 22519384

[fsn34762-bib-0041] Mohammadifard, N. , A. Mahdavi , A. Khosravi , A. Esmaillzadeh , A. Feizi , and N. Sarrafzadegan . 2021. “Salt Intake and Its Sources in Children, Adolescents and Adults in the Islamic Republic of Iran.” Eastern Mediterranean Health Journal 27, no. 3: 279–286. 33788217 10.26719/2021.27.3.279

[fsn34762-bib-0042] Moslemi, M. , M. Kheirandish , N. Mazaheri , et al. 2020. “National Food Policies in the Islamic Republic of Iran Aimed at Control and Prevention of Noncommunicable Diseases.” Eastern Mediterranean Health Journal 26: 1556–1564. 33355396 10.26719/emhj.20.024

[fsn34762-bib-0043] Naghavi, M. , F. Abolhassani , F. Pourmalek , et al. 2009. “The Burden of Disease and Injury in Iran 2003.” Population Health Metrics 7, no. 1: 1–21. 19527516 10.1186/1478-7954-7-9 PMC2711041

[fsn34762-bib-0044] Navidghasemizad, S. , J. Hesari , P. E. R. Saris , and M. R. Nahaei . 2009. “Isolation of Lactic Acid Bacteria From Lighvan Cheese, a Semihard Cheese Made From Raw Sheep Milk in Iran.” International Journal of Dairy Technology 62, no. 2: 260–264. 10.1111/j.1471-0307.2009.00462.x.

[fsn34762-bib-0045] NCCO Food , and Ministry Of Health Malaysia . 2010. Malaysian Dietary Guidelines. Malaysia: National Coordinating Committee on Food and Nutrition, Ministry of Health.

[fsn34762-bib-0046] NNFTRI . 2017. National Program of Monitoring of Nutritional Risk Factors in Iran Industrial and Guild Foods, 2016. Tehran, Iran: National Nutrition and Food Technology Institute, Shahid Beheshti University of Medical Sciences.

[fsn34762-bib-0047] Noori, A. , F. Keshavarzian , S. Mahmoudi , M. Yousefi , and L. Nateghi . 2013. “Comparison of Traditional Doogh (Yogurt Drinking) and Kashk Characteristics (Two Traditional Iranian Dairy Products).” European Journal of Experimental Biology 3, no. 6: 252–255.

[fsn34762-bib-0048] Nosratinia, N. , S. Azadnajafabad , M. Masinaei , et al. 2024. “Salt Intake Among Iranian Population: National and Subnational Report From STEPS 2021.” 10.1002/fsn3.71399 PMC12750446 41476714

[fsn34762-bib-0049] Nottagh, S. , J. Hesari , S. H. Peighambardoust , R. Rezaei‐Mokarram , and H. Jafarizadeh‐Malmiri . 2020. “Effectiveness of Edible Coating Based on Chitosan and Natamycin on Biological, Physico‐Chemical and Organoleptic Attributes of Iranian Ultra‐Filtrated Cheese.” Biologia 75, no. 4: 605–611. 10.2478/s11756-019-00378-w.

[fsn34762-bib-0050] Omrani Khiabanian, N. , A. Motamedzadegan , S. Naghizadeh‐Raisi , and M. Alimi . 2020. “Chemical, Textural, Rheological, and Sensorial Properties of Whey‐Less Feta Cheese as Influenced by Replacement of Milk Protein Concentrate With Pea Protein Isolate.” Journal of Texture Studies 51, no. 3: 488–500. 10.1111/jtxs.12508. 31994729

[fsn34762-bib-0051] Organization, W. H . 2011. Causes of Death 2008: Data Sources and Methods. Geneva: World Health Organization.

[fsn34762-bib-0052] Pourkhajoei, S. , V. Yazdi‐Feyzabadi , M. Amiresmaeili , N. Nakhaee , and R. Goudarzi . 2022. “Mean Population Salt Intake in Iran: A Systematic Review and Meta‐Analysis.” Health Science Reports 5, no. 6: e855. 10.1002/hsr2.855. 36226320 PMC9531774

[fsn34762-bib-0053] Raymond, J. L. , and K. Morrow . 2022. Krause and Mahan's Food and the Nutrition Care Process, 16e, E‐Book. Saunders Philadelphia: Elsevier Health Sciences.

[fsn34762-bib-0054] Rezac, S. , C. R. Kok , M. Heermann , and R. Hutkins . 2018. “Fermented Foods as a Dietary Source of Live Organisms.” Frontiers in Microbiology 9: 396129. 10.3389/fmicb.2018.01785. PMC6117398 30197628

[fsn34762-bib-0055] Rezaei, S. , Z. Mahmoudi , A. Sheidaei , et al. 2018. “Salt Intake Among Iranian Population: The First National Report on Salt Intake in Iran.” Journal of Hypertension 36, no. 12: 2380–2389. 30005027 10.1097/HJH.0000000000001836

[fsn34762-bib-0056] Ribeiro, B. D. , R. P. do Nascimento , K. S. Pereira , and M. A. Z. Coelho . 2018. Microbiologia Industrial: Alimentos. Vol. 2. Brazil: Elsevier.

[fsn34762-bib-0057] Rosma, A. , T. Afiza , W. Wan Nadiah , M. Liong , and R. Gulam . 2009. “Short Communication Microbiological, Histamine and 3‐MCPD Contents of Malaysian Unprocessed ‘Budu’.” International Food Research Journal 16: 589–594.

[fsn34762-bib-0058] Roustaee, R. , H. Eini‐Zinab , D. Ghodsi , et al. 2024. “A 30‐Year Trend of Dairy Consumption and Its Determinants Among Income Groups in Iranian Households.” Frontiers in Public Health 12: 1261293. 10.3389/fpubh.2024.1261293. 38425466 PMC10903262

[fsn34762-bib-0059] Rysová, J. , and Z. Šmídová . 2021. “Effect of Salt Content Reduction on Food Processing Technology.” Food 10, no. 9: 2237. 10.3390/foods10092237. PMC8469246 34574347

[fsn34762-bib-0060] Savaiano, D. A. , and R. W. Hutkins . 2021. “Yogurt, Cultured Fermented Milk, and Health: A Systematic Review.” Nutrition Reviews 79, no. 5: 599–614. 32447398 10.1093/nutrit/nuaa013 PMC8579104

[fsn34762-bib-0061] Sebranek, J. G. 2015. “An Overview of Functional Non‐Meat Ingredients in Meat Processing: The Current Toolbox.” In Paper presented at the In Proceedings of the American Meat Science Association, 68th Annual Reciprocal Meat Conference, 14–17. Lincoln, NE: University of Nebraska‐Lincoln. Accessed 5 August, 2021. http://www.meatscience.org/docs/default‐source/publications‐resources/rmc/2015/09_sebranek_f.pdf.

[fsn34762-bib-0062] Shahrajabian, M. H. , and W. Sun . 2023. “Kashk and Doogh: The Yogurt‐Based National Persian Products.” Current Nutrition & Food Science 19, no. 9: 922–927.

[fsn34762-bib-0063] Shiroodi, S. G. , M. A. Mohammadifar , E. G. Gorji , H. Ezzatpanah , and N. Zohouri . 2012. “Influence of Gum Tragacanth on the Physicochemical and Rheological Properties of Kashk.” Journal of Dairy Research 79, no. 1: 93–101. 10.1017/S0022029911000872. 23171586

[fsn34762-bib-0064] Soltani, M. , and N. Güzeler . 2013. “The Production and Quality Properties of Liquid Kashks.” Gida 38: 1–7.

[fsn34762-bib-0065] Song, X. , J. Fu , Q. Ding , et al. 2024. “Antihypertensive Effects of the Limosilactobacillus Reuteri Z09 and *Lactobacillus helveticus* Z11 Fermented Milk in Spontaneously Hypertensive Rats.” International Journal of Dairy Technology.

[fsn34762-bib-0066] Spieldenner, J. , and K. van der Horst . 2018a. “Reformulating Food Products for Improved Nutrition.” Sight and Life 32, no. 1: 67–71.

[fsn34762-bib-0067] Spieldenner, J. , and K. van der Horst . 2018b. “Reformulating Food Products for Improved Nutrition or: How to Improve Processed Foods Quietly.” Sight and Life 32: 67–71.

[fsn34762-bib-0068] Stones, M. 2014. “Beware Food Safety Impact of Reformulation.” Inside Food & Drink Manufacturing. https://www.foodmanufacture.co.uk/article/2014/10/22/food‐safety‐could‐be‐threatened‐by‐reformulation.

[fsn34762-bib-0069] Taheur, F. B. , A. Chahbani , C. Mansour , et al. 2023. “Functional Properties of a Kefir‐Based Probiotic Dairy Product Enriched With Red Prickly Pear ( *Opuntia dillenii* ) Powder.” Journal of Food Measurement and Characterization 17, no. 6: 6522–6535.

[fsn34762-bib-0070] Taylor, R. S. , K. E. Ashton , T. Moxham , L. Hooper , and S. Ebrahim . 2011. “Reduced Dietary Salt for the Prevention of Cardiovascular Disease: A Meta‐Analysis of Randomized Controlled Trials (Cochrane Review).” American Journal of Hypertension 24, no. 8: 843–853. 21731062 10.1038/ajh.2011.115

[fsn34762-bib-0071] Tewari, S. , J. David , and A. Gautam . 2019. “A Review on Probiotic Dairy Products and Digestive Health.” Journal of Pharmacognosy and Phytochemistry 8, no. 3: 368–372.

[fsn34762-bib-0072] Wallis, K. , S. Chapman , and B. Campden . 2012. Current Innovations in Reducing Salt in Food Products. UK: Food & Health Innovation Service, Campden BRI.

[fsn34762-bib-0073] WHO . 2010. World Health Organization. Creating an Enabling Environment for Population‐Based Salt Reduction Strategies: Report of a Joint Technical Meeting Held by WHO and the Food Standards Agency, United Kingdom, July 2010. World Health Organization.

[fsn34762-bib-0074] WHO . 2013. A Global Brief on Hypertension: Silent Killer, Global Public Health Crisis. World Health Organization.

[fsn34762-bib-0075] WHO . 2022. Universal Salt Iodization and Sodium Intake Reduction: Compatible, Cost‐Effective Strategies of Great Public Health Benefit. Geneva: World Health Organization.

[fsn34762-bib-0076] Wilson, R. , E. Komitopoulou , and M. Incles . 2012. “Evaluation of Technological Approaches to Salt Reduction.” Leatherhead Food Research. https://www.fdf.org.uk/resources/salt_reduction_2012.pdf.

[fsn34762-bib-0077] World Health Organization . 2012. Guideline: Sodium Intake for Adults and Children. World Health Organization. 23658998

[fsn34762-bib-0078] World Health Organization and Food and Agriculture Organization . 2011. Codex Alimentarius, Milk and Milk Products. Rome: World Health Organization and Food and Agriculture Organization of the United Nations.

[fsn34762-bib-0079] Wyness, L. A. , J. L. Butriss , and S. A. Stanner . 2012. “Reducing the Population's Sodium Intake: The UK Food Standards Agency's Salt Reduction Programme.” Public Health Nutrition 15, no. 2: 254–261. 10.1017/S1368980011000966. 21729460

[fsn34762-bib-0080] Zhu, J. , Y. Chen , C. Lv , W. Wu , and S. Qin . 2019. “Study on Optimization of Removing Cadmium by Lactobacillus Fermentation and Its Effect on Physicochemical and Quality Properties of Rice Noodles.” Food Control 106: 106740. 10.1016/j.foodcont.2019.106740.

[fsn34762-bib-0081] Zonoubi, R. , and M. Goli . 2021. “The Effect of Complete Replacing Sodium With Potassium, Calcium, and Magnesium Brine on Sodium‐Free Ultrafiltration Feta Cheese at the End of the 60‐Day Ripening Period: Physicochemical, Proteolysis–Lipolysis Indices, Microbial, Colorimetric, and Sensory Evaluation.” Food Science & Nutrition 9, no. 2: 866–874. 10.1002/fsn3.2050. 33598170 PMC7866566

